# Australian School Stakeholders’ Perceived Strategies for Preventing Adolescent Obesity

**DOI:** 10.3390/ijerph18179387

**Published:** 2021-09-06

**Authors:** Kakale Buru, Theophilus I. Emeto, Aduli E. O. Malau-Aduli, Bunmi S. Malau-Aduli

**Affiliations:** 1 College of Medicine and Dentistry, James Cook University, Townsville, QLD 4811, Australia; kay.buru@my.jcu.edu.au; 2 College of Public Health, Medical and Veterinary Sciences, James Cook University, Townsville, QLD 4811, Australia; theophilus.emeto@jcu.edu.au (T.I.E.); aduli.malauaduli@jcu.edu.au (A.E.O.M.-A.)

**Keywords:** adolescent obesity, stakeholders, interventions, physical activity

## Abstract

Adolescent obesity is a complex multifactorial disease with a combination of environmental, behavioral, psychosocial, biological, cultural and genetic determinants. It remains a global public health issue that presents a major challenge to chronic disease prevention and health into adulthood. Schools have a rich opportunity to improve youth health and tackle obesity, yet they face barriers in fulfilling this function. This study investigated school stakeholders’ beliefs and perceptions of the barriers and enablers currently experienced by schools, as well as their recommendations towards preventing adolescent obesity. A sequential explanatory mixed-methods study design was utilised with surveys administered for the quantitative phase and individual interviews for the qualitative phase. Descriptive statistics and inductive thematic analyses were utilised for the survey and interview data, respectively. Triangulation of findings from the quantitative and qualitative phases aided in the better understanding and integration of the overall results. In total, 60 school stakeholders (52 subject teachers, 3 senior teachers and 5 heads of department) from both independent and public high schools in Queensland, Australia responded to the survey, while 14 respondents participated in the interviews. The main perceived causes of obesity were poor eating habits and sedentary lifestyle. Highlighted barriers were busy timetables, shortage of trained staff and funding, lack of robustness in the introduction and implementation of school interventions and insufficient motivation of learners to participate in obesity prevention programs. Enabling factors included parental support, easy access to fitness equipment during recess, supportive government policies, provision of healthier school tuck shop menu options and elimination of sugary drinks from vending machines. A model for the prevention of adolescent obesity was developed based on participants’ perceptions. Tripartite collaboration between the school, government and parents was perceived as fundamental to preventing adolescent obesity. Strategies targeting nutrition, physical activity and overall health, including parental education on health, formal health talks in schools by health professionals and better-targeted advertisement encouraging healthy lifestyle choices, were identified as essential for improved adolescent health outcomes.

## 1. Introduction

Obesity is one of the leading risk factors for global mortality and the exponential increase in the prevalence of obesity among children and adolescents has been projected to reach 91 million by 2025 [1]. Published literature indicates that adolescents with obesity have higher chances of progressing to persistent obesity in adulthood [2]. Wadman et al. [3] reported that in the USA, 77% of patients hospitalized due to COVID-19 complications had conditions attributable to overweight and obesity. This implies that obesity is both a major risk factor that may be linked to other physical health conditions (such as diabetes and heart diseases) and a potential effect of lockdown [4]. In Australia, one in every four adolescents is overweight [2], making Australia one of the top 10 countries with the highest proportion of adolescents with obesity [5]. The prevalence of obesity may even be higher in recent times due to inactivity among adolescents worldwide, resulting from COVID-19 restrictions [6], a condition now referred to as ‘Covibesity’ [7]. It is therefore paramount to focus on actions that could possibly prevent and reduce the prevalence of obesity in adolescents [8,9,10].

Obesity is associated with increased energy intake and decreased energy expenditure [10]. It is a multifaceted chronic condition with several contributing factors, including medical illnesses, biological risk factors, genetic disorders, eating disorders [10,11], health literacy, cultural background, socioeconomic status (SES) and numerous environmental influences [12,13]. Adolescent obesity increases the risk of chronic disease development into and throughout adulthood [14]. Obesity in adolescents impacts all major organ systems and often contributes to morbidity [15,16]. Obesity prevalence in adolescents is also exacerbated by differences in ethnic and genetic backgrounds, which affect body composition and fat distribution [17] and cultural body image standards [18].

The current social climate, particularly within high-income countries, with emphasis placed on health messaging and weight loss within society, creates a stressful state for adolescents with obesity due to weight stigma [19,20]. Adolescents who experience stress related to social ostracization are more likely to rely on food-related coping mechanisms [21]. For example, adolescents with obesity may experience teasing and bullying, which can lead to isolation and inability to make friends [22]. Isolation can negatively affect the mental well-being of adolescents due to deprivation of social interaction needed at this stage of development [23]. The interplay between obesity and psychosocial health may lead to increased levels of stress, depressive symptoms and reduced resilience [11,23]. This is further confounded with SES, which is one of the most potent indicators of overall health [13,24]. Youths from low socio-economic backgrounds tend to have higher rates of obesity compared to other groups [13].

The school is perceived to be at a vantage point in the prevention of adolescent obesity because it provides an opportunity for longer contact hours during school days for successful implementation of interventions [25,26,27]. It is therefore important to consider maximizing the use of school environments in preventing adolescent obesity. The school environment can promote physical activity and healthy eating [10] and has been described as the perfect nexus for teachers, parents and other stakeholders to modify and implement lifestyle, behavioural and nutritional interventions to impede the progress of childhood and adolescent obesity [11,28]. Lambrinou et al. [28] provided a detailed review of effective strategies for obesity prevention via school-based, family-involved interventions and the advantages of the school environment. Nonetheless, research findings indicate that despite the advantages associated with the school environment, the benefits of school-based interventions are questionable and generalized recommendations are difficult to extract and extrapolate [29,30,31]. Studies have mostly focused on stakeholder views on the primary school role in preventing obesity [26,32]. Within the Australian context, school-based prevention programs are not widely implemented in high schools [33]. A recent review reported weak evidence for the efficacy of the interventions and programs identified in Australian high schools, particularly because of the weak link between teacher involvement and modification of the food environment during the interventions [34]. Additionally, most of the Australian adolescent obesity research emanated from the State of New South Wales and no studies were nationwide [34]. It is therefore important to reassess the role of the school in the prevention of adolescent obesity. More importantly, due to the many health implications associated with obesity, it is expedient to target preventive measures rather than a cure. Exploration of school stakeholders’ opinions about current priorities, barriers and enabling factors would be beneficial in the global effort to prevent adolescent obesity. Additionally, recommendations from school stakeholders are key to future planning and implementation of effective policies and intervention programs [29]. This study therefore sought to investigate Queensland, Australia school stakeholders’ beliefs and perceptions of the barriers and enablers currently experienced by schools and their recommendations for preventing adolescent obesity. The study also aimed to develop a reliable adolescent obesity prevention model based on the findings.

## 2. Materials and Methods

Ethics approval (Approval number: H7966) for this study was obtained from the James Cook University’s Human Research Ethics Committee (HREC).

### 2.1. Study Design

A sequential explanatory mixed methods study design was utilised. This design employs a methodical integration of quantitative and qualitative research approaches within a single study to offer detailed explanation of results [35]. Findings from the quantitative phase comprising online surveys aided the development of the interview questions for the qualitative phase [36]. The inherent weaknesses due to bias in both quantitative and qualitative approaches were addressed by triangulating findings from both phases to uncover the best possible explanations for the observed phenomenon [35].

### 2.2. Quantitative Phase

For the quantitative phase, data were obtained from responses to online survey questions on the perceptions and beliefs of school stakeholders from Queensland Education towards the prevention of adolescent obesity.

#### 2.2.1. Participants

Study participants included school stakeholders (teachers and senior administrators) from both independent and public Queensland high schools who are certified employees of Queensland Education. Primary school stakeholders were excluded from participation. Information about the study was communicated through paid adverts to different school stakeholder online networks/groups via social media platforms including Facebook, WhatsApp and LinkedIn. Prospective participants were provided with a link to the survey. Snowballing [37] and follow-up phone calls were made to those who indicated interest in the study to maximize participation. The survey was administered electronically to participants via Qualtrics XM, Utah, United States. An information sheet that stated the purpose and confidentiality protocols for the study was provided to prospective participants and informed consent was obtained prior to completion of the survey. 

#### 2.2.2. Data Collection Instrument

A questionnaire comprising closed and open-ended questions was used. The survey questions were categorized into six (6) sections that examined participants’ background information, their beliefs, attitudes and perceptions about available anti-obesity policies as well as the barriers and enablers of school-based prevention programs. The survey questions were adapted from two previous studies [38,39]. The questions on beliefs, attitudes and perceptions about obesity were adapted from the study by Price et al. [39], and the questions on anti-obesity policies and school-based prevention programs were adapted from the study by Kennedy et al. [38]. The survey instrument was pilot tested and there was no need to revise any of the questions. The last question in the survey was used to identify those who had interest in participating in the follow-up individual interviews. However, the quantitative findings and the participants’ demographic details were utilised in purposively selecting the interview participants to ensure involvement of all participant groups until data saturation was reached [40].

#### 2.2.3. Data Analysis

Quantitative data were analysed using SPSS version 27. Descriptive statistics in the form of frequencies and percentages were used to identify most occurring perceptions, beliefs and attitudes, barriers and enablers, types of policies and prevention programs used in schools.

### 2.3. Qualitative Phase

#### 2.3.1. Data Collection

Responses from the quantitative phase were used to guide the development of semi-structured open-ended interview questions for the qualitative phase. The interviews were conducted using Zoom cloud meeting between December 2020 and February 2021. Each interview session lasted approximately 30–60 min. Interviews were recorded and transcribed for textual analysis. This phase of the study was intended to foster in-depth understanding of the participants’ perceptions on the main enablers and barriers to prevention of adolescent obesity, and recommendations on what they perceive would work best within their context in the prevention of adolescent obesity. The semi-structured interview guide used for this phase of the study is provided in Appendix A.

#### 2.3.2. Data Analysis

The qualitative data were analysed using NVivo 12 plus, guided by inductive thematic analysis approach [40]. Coding and analysis of interview data were performed at two levels: within each case and across the cases [41]. Analysis of the interview transcripts included multiple readings to aid the identification of emerging themes [42]. Transcripts were explored for meaning in participants’ words and language. During the iterative coding stage, transcripts were independently assessed by two researchers with different professional backgrounds to widen perspectives (K.B. and B.S.M.-A). Identified themes as well as patterns of similarities and divergence were discussed and confirmed, with discrepancies resolved in a consensus meeting. The trustworthiness and credibility of findings were established through member checking, and cross matching of emerging themes by the researchers [40]. Verbatim quotations are presented to illustrate the emerging themes.

## 3. Results

### 3.1. Quantitative Phase

Overall, 90 school stakeholders consented to participate in this phase of the study. However, only 60 of them completed the online survey, with a 67% response rate. Table 1 presents the profile of the survey respondents. There were 60 participants, and 47 (78%) of them were females. Respondents’ roles were heads of department (5, 8%), senior teachers (3, 5%) and subject teachers (52, 87%). Most respondents were from public schools (70%), worked full-time (67%) and had a Bachelor’s degree (63%), while 32% had a Master’s degree or higher.

Table 2 portrays participants’ top three agreement responses and the least agreed response in relation to their perceptions and beliefs about the causes, enabling and hindering factors of adolescent obesity and the health priorities considered in their schools.

#### 3.1.1. Participants’ Perception of Obesity

Table 2 depicts that majority (98%) of the participants indicated poor eating behaviour as the major cause of adolescent obesity, followed by sedentary lifestyle and excessive calorie consumption (93%). Peer pressure was rated as the least likely (39%) possible cause of adolescent obesity.

#### 3.1.2. Participants’ Beliefs

Most participants (87%) believed that adolescent obesity is becoming more prevalent and that having a healthy weight is very important for adolescents. About two-thirds (68%) of the participants believed that adolescent obesity is a significant cause of peer rejection, and only 3% believed that only youths who are likely to succeed in a weight loss program should be part of a treatment plan (see Table 2).

#### 3.1.3. Perceived Enabling Factors in Adolescent Obesity Prevention

When asked what they thought could be enabling factors for schools in supporting the prevention of adolescent obesity, 86% of the participants indicated parental support, regular evaluation of available intervention programs and elimination of ‘junk’ food and beverage machines from schools. Availability of low-calorie healthy lunches during lunch hour was also considered a key factor for preventing adolescent obesity by 86% of the participants. Supportive government policies and ease of access to fitness equipment during recess were also perceived by 78% of the respondents to be enabling factors. Two-thirds of the participants (65%) felt that community involvement could also help in enabling the school to prevent adolescent obesity (see Table 2).

#### 3.1.4. Perceived Barriers to Adolescent Obesity Prevention

The majority of the participants (82%) felt that busy school timetable was the main barrier to adolescent obesity prevention, followed by shortage of trained staff and insufficient funding (78%), the absence of thoroughly implemented intervention plans (75%) and insufficient motivation of learners to participate in obesity prevention programs (70%). Only 32% perceived short school day as a barrier (see Table 2).

#### 3.1.5. Health Priorities in Schools

The study findings in Table 2 show that emotional and mental health were the main health priorities of the school as indicated by 90% of the participants, followed by relational and social skills (48.3%). Two-fifths of the participants (40%) reported physical fitness as a priority and only one participant indicated that tobacco use was the main health focus at their school.

Table 3 presents the interventions and strategies currently used by schools for physical health and wellness. The predominant (78%) strategy used was health and physical education (HPE), 45% of the participants indicated that there were other forms of physical activity programs offered besides HPE. Nutrition education and promotion was reported by 35% of the participants as a strategy used by their school. Nutrition standards for meals (12%) and BMI tracking/reporting (2%) seemed to be unpopular strategies in schools.

### 3.2. Qualitative Phase

As shown in Table 4, 14 school stakeholders, who were predominantly females (*n* = 11) and between the ages of 25 and 60 years, participated in the interviews. Eight (*n* = 8) of the participants were teachers in public state high schools, while six were from independent high schools. The participants had predominantly teaching roles, all of them had at least a first degree and between 3 and 50 years of teaching experience.

**Figure 1:**
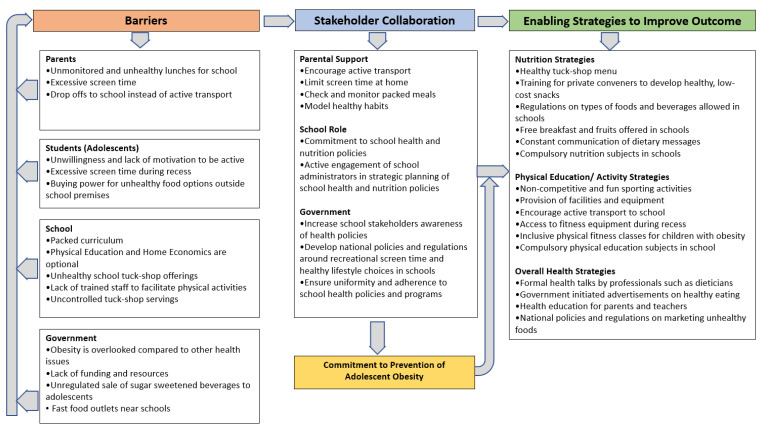
Model for the prevention of adolescent obesity representative of Queensland school stakeholders’ perceptions.

Thematic analysis of the interview data presented three emergent themes: barriers schools encounter in the prevention of obesity. need for stakeholder collaborations and enabling strategies to improve outcomes. These themes are presented with verbatim illustrative quotes, and each quote is depicted using participant’s name and school type (PS = public school and IS = independent school). The study findings were used to develop a reliable model for the prevention of adolescent obesity (see Figure 1). Table 5 depicts how the quantitative results were clarified and confirmed by the participants’ responses in the qualitative phase of the study.

#### 3.2.1. Barriers Schools Encounter in the Prevention of Obesity

Participants indicated that the prevention of adolescent obesity is usually hindered by four (4) major stakeholder groups, namely parents, students, the school and the government.

##### Barriers Associated with Parents

Participants felt some parents encouraged their children and wards to engage in unhealthy eating habits and lifestyle. Parents were considered too busy to prepare and pack healthy lunches for their teenage children. Participants also reported that adolescents indulge in excessive screen time more at home, where they are usually unmonitored by parents.

*‘* *If parents habitually indulge in junk food, children are going to mimic that, and they are going to think it’s fine and the pattern will be hard to remedy in later life.’ Ruth, IS*

*‘Ideally parents should have a great contribution because they are the ones who buy the food for the household, which the students are eating two-thirds of the time, with only one main meal a day at school. But a couple of things come into play, a lot of parents are time poor. I think it’s much more a thing that parents prepare lunchboxes for little ones, but once they [kids] get to high school, it stops.’ Jessica, PS*

*‘I think that unmonitored screen time mostly is even more rampant at home than at school, because you know these kids sit down and play video games and go on social media or whatever it is [called].’ Jesse, IS*

##### Barriers Associated with Students

Students’ unwillingness and lack of motivation to be active, especially if they are overweight, were seen as major obstacles. Students were also reported to spend enormous amount of time on electronic devices during recess instead of engaging in physical activities.

*‘Students shy away from activities like swimming because they feel ashamed of being seen in swimmers if they are overweight or obese’, Sonia, IS*

*‘During the breaks you find every child looking at a screen, whether sitting in a group or alone [when] they could spend more time talking and engaging in other activities’, Raphael, PS*

*‘There’s not much that would make the children think it’s worth their while to participate unless they really enjoyed healthy eating or they enjoyed physical activity, before they came to the school’, Janelle, IS.*

The participants also felt that adolescents often had part-time jobs that gave them access to pocket money, which gave them ‘buying power’ to engage in unhealthy food choices as observed in the foods they ordered from the canteen or the lunches from home.

*‘In the school canteen, students pass through a line and there’s a section of cold food, [another] section of hot food, and they get given whatever they want, so students could choose six pieces of pizza, if they wanted to and get handed that with no problem [monitoring]’ Angelica, PS*

*‘Quite often the kids have part time jobs, or they’ve got pocket money, and they can buy whatever they choose to.’ Jessica, PS*

*‘We can’t really control the food that they bring in, or what they choose to eat, or the choices they make once they leave the school grounds, either.’ Janelle, IS*

##### Barriers Associated with the School

A major barrier identified at the school level was the busy or ‘crowded curriculum’, which makes it challenging to offer physical activities regularly.

*‘Doing those kinds of activities with students outside the curriculum is a challenge because the curriculum is too saturated already.’ Sonia, PS*

*‘The push in the curriculum to do this and this and this and this, in addition to the core subjects. And so, those blocks of time that used to be for physical education or Wednesday afternoon sports for 70 min have been eroded.’ Jessica PS*

Schools’ close proximity to fast food outlets was another identified barrier. Unhealthy school tuck shop menu where serves and portions are not controlled for students was also seen as a major drawback, particularly in public schools. To help students make better food choices, participants felt subjects like Home Economics and HPE could be made compulsory.

*‘Even if the school only provides healthy options, some of them [students] will sneak down to the service station and get soft drink and stuff like that or bring it from home.’ Jessica, PS*

*‘The number of healthy items they sell at the school tuck shop is quite low in comparison to the number of unhealthy items.’ Angelica, PS*

*‘I know there’s government policies that they’ve implemented as to what tuck shop sells but I’m not aware of the specific rules.’ Samantha, PS*

*‘Home Economics, HPE and food and nutrition subjects should be made compulsory subjects in school from grade seven to grade 12.’ Sage, PS*

Lack of trained staff to facilitate physical activities was also identified as a barrier, especially in public schools. Interestingly, most of the participants indicated that there were inconsistencies across Queensland schools, and they were unaware of available national policies on prevention of adolescent obesity in schools.

*‘I took my Year 7s down to the oval for HPE and that was really awful. I said I wouldn’t take them again because I had kids rolling around the hill and then somebody got kicked and started crying, and then somebody else got pushed over and started crying. And I didn’t really have the experience to manage that situation very well so we’re not going down to the oval again.’ Rebecca, PS*

*‘In Queensland it’s up to the individual school, except if there is a system I am not aware of. I wouldn’t say that if we’re following clear intervention policies that I have been made aware of them’, Janelle, IS*

##### Barriers Associated with the Government

The major barrier identified at this level was laxity on the part of the government in implementing policies that help curb adolescent obesity.

*‘‘We still see a lot of food that are being sold that are not very healthy in the tuck shops, so we need stricter guidelines and policies from the government and making sure that all schools, not just some schools, all schools [adhere] to the policies where they don’t sell unhealthy foods in the tuck shop. They [government] also make home economics policy where all students learn about nutrition and about healthy eating habits.’ Sonia, IS*

Participants felt that the lack of funding and resources, particularly in public schools, make it challenging to run activities for the prevention of adolescent obesity compared to other health issues like mental health.

*‘I think particularly public schools, which is where I’ve had most of my experience, are trying to do the best we can with very limited funding. I think we’re trying but there’s definitely work still to be done and there’s only so much you can do without additional resources to help.’ Janelle, IS*

#### 3.2.2. Stakeholder Collaboration

Participants indicated that collaboration between three major stakeholders—parents, the school and the government, is needed. They stressed that it is not the sole responsibility of the school to facilitate prevention of adolescent obesity.

##### Parental Support

Participants indicated that parents, as a stakeholder group, could encourage active transport to school and sign up their children for sporting activities at school. Responses also pointed to limiting screen time at home, checking and monitoring packed meals for school lunches. Parent educating and modelling healthy habits was emphasized as a way of ensuring that children do not mimic unhealthy habits

*‘Parents can also show support by making sure their children play school sports or signing them up to a sporting club as well.’ Vanya, PS*

*‘I think parental support is very important, because formative years of a child begins at home; particularly limiting screen time, nutrition, what they eat.’ Raphael, PS*

*‘Parental role in educating and modelling good eating habit is vital in encouraging a healthy lifestyle for their children, and other aspects such as their diet, to support prevention of adolescent obesity.’ Jesse, IS*

##### School Role

Participants indicated that the school could play a more active role in this collaboration by ensuring that there is whole of organization commitment to health and nutrition policies and programs, participation in strategic planning and decision-making to advance the prevention of adolescent obesity. Participants expressed the need for the school to educate and empower parents to make better lifestyle choices for themselves and their children.

*‘But what we really need to be doing as a school is giving education and empowering parents to make better choices and backing them up on those choices.’ Angelica, PS*

*‘I think educating parents can be very helpful. You know, like we get news items going up from school and things of concern. It goes to all parents, so they don’t feel like they are being targeted or victimized or in any way ostracized.’ Raphael, PS*

They also emphasized the importance of educating students and promoting compulsory sporting/physical activities in all schools.

*‘In terms of promoting physical activity, some schools I’ve been at in the past have made sports compulsory. At my current school, the kids didn’t do sports for the past year 10. So, I had to do PE [with the students] at lunchtime.’ Sage, PS*

*‘Wherever possible in our syllabus and curriculum if you can talk about that. That’s helpful as well. I mean I had the opportunity to do that when I taught HPE. So, I used that as a platform to talk about the importance of a good diet.’ Raphael, PS*

*‘Participation is not that optional. Everyone is supposed to participate in extracurricular activities.’ Hebron, IS*

##### Government Role

Participants indicated that the government could enact policies that ensure uniformity in educational programs and policies across schools and implement phone usage policy to regulate recreational screen time in schools.

*‘The government is the one that streamlines the curriculum, and provides funding for schools, obviously, the government can play a good role to support schools’, Jesse, IS.*

*‘The government should come up with strict policy on what the Tuck Shop can sell and what they can’t.’ Sonia, IS*

*‘To address screen time, I think there should be more firm rules around that. My opinion is that the phones should be banned for the entire day at school unless emergencies, but I know there’s a lot of debate in the education community about that.’ Janelle, IS*

Participants pointed out that the government should reintroduce programs like ‘Smart Moves’, which actually helped to promote and enforce physical activity programs, as well as acceptable food policies that clearly state what types of food and drinks are allowed or prohibited in schools. Participants also felt that the government could enforce media advertisement of healthy food instead of junk food and subsidize or give free vouchers for sporting activities to make them affordable for young adults.

*‘The government could reintroduce Smart Moves, which actually helped to promote physical activities like doing sports and exercise.’ Sage, PS*

*‘I think a lot of that is dictated by government regulations. I remember a few years ago they categorized food into red and yellow and green. I think those colour codes meant red was the junk food and you could have the occasional red day, and green was healthy foods. Yellow was not as healthy as green but not as bad as the red, and I think you’re allowed to sell a certain amount of red, but not a lot. And you could have the occasional red day where there would be more junk food available. I’m not sure if it’s followed in this school but I’m pretty sure that there has to be regulations they follow as to what they sell at the school, as well as for prevention of obesity.’ Samantha, PS*

*‘The government can pay the media to advertise healthy food instead of junk food.’ Ruth, IS*

#### 3.2.3. Enabling Strategies to Improve Health Outcomes

Participants in this study recommended standardized implementation of enabling strategies. Major areas of focus included nutrition, physical education activities and overall health and well-being.

##### Nutrition Strategies

The nutrition strategies related majorly to tuck shop menu. Participants suggested a move towards national implementation of food and beverage policy within schools to monitor types of food and drinks students can/cannot bring to school. Other nutrition-related strategies to improve outcome included the provision of free fruits and breakfast to students, compulsory nutrition subjects and constant communication of dietary messages within schools.

*‘The school administration should look into what food options are healthy for the tuck shop.’ Hebron, IS*

##### Physical Education/Activity Strategies

Participants recommended social, enjoyable and non-competitive sporting activities to improve outcomes in adolescent obesity prevention. Government funding and provision of accessible fitness equipment/facilities would also improve outcomes.

*‘I think social sport, that’s compulsory and all it’s there for all levels, not necessarily competitive sport but more like social sport would be great.’ Chantelle, IS*

*‘Students have to choose across the four terms at least something to do with physical activity, but that’s not the system at the moment as students just get to choose whatever they want to’, Angelica, PS*

##### Overall Health and Well-Being Strategies

Participants pointed out that formal health talks ‘with health experts like dieticians and nutritionists can be organized to educate students and their parents about healthy lifestyle. Another strategy identified was targeted government-sponsored advertisements on healthily eating.

*‘I think definitely speaking about these things and holding sessions where health experts like dieticians and nutritionists can come in and educate the students,’ Ruth, IS*

*‘I think government plays a part in making sure that advertising continues to happen regarding what a healthy diet looks like, and how much physical activity people should be getting.’ Vanya, PS*

Commitment to prevention of adolescent obesity requires tripartite collaboration between the school, parents and government to adequately tackle the identified barriers and enhance health outcomes.

## 4. Discussion

This study investigated school stakeholders’ beliefs and perceptions about the barriers and enablers currently experienced by schools and their recommendations for preventing adolescent obesity. Our findings are summarized into a model for the prevention of adolescent obesity in schools (Figure 1). Barriers currently experienced by the school towards preventing adolescent obesity were explored and classified into four stakeholder levels: the students, parents, school and government. Our findings show that stakeholder collaboration is the missing link that is essential to dealing with adolescent obesity. Recommendations of what the parents, the school and the government can do in their respective roles to help prevent adolescent obesity were identified. The participants emphasized the adoption of strategies that have the potential to increase physical activity, reduce screen time and promote healthy eating habits among adolescents. Overall, the findings show that the school cannot deal with the burden of adolescent obesity alone, and that complimentary collaborative efforts from all three major stakeholder groups (the school, parents and government) are required to combat adolescent obesity.

The school has been identified as an ideal place for adopting and implementing strategies to tackle adolescent obesity, because majority of adolescents attend school, providing an opportunity for longer contact periods during school days [25,26,27,28]. However, it is quite evident in this study that obesity prevention efforts will be futile if the adolescents themselves are not motivated. The participants made a general observation that adolescents are often unwilling and uninterested in taking part in physical activities. This finding confirms a recent report that emphasizes adolescents’ indifference to physical activity if it competes with other interests such as hanging out with friends or doing screen-based activities [12,43,44].

Nonetheless, if the teachers are unaware of relevant policies and have insufficient skills needed to facilitate outdoor activities, they will not feel confident to assist with promoting activities that aid prevention of adolescent obesity. It is evident in this study that teachers lack skills in facilitating outdoor physical activities and are also not aware of what policies to follow for such activities. Teachers need to be aware of current policies and actively engage in strategic planning and decision-making processes within the school for better implementation of policies [45]. School administrators are encouraged to involve teachers in the development of such policies and frequently give policy reminder talks at least once per semester to foster awareness and facilitate implementation of the strategies set in the policies.

Our findings that school tuck shops seem to be encouraging unhealthy eating habits among adolescents are consistent with the report by Ronto and colleagues [46] that an unhealthy tuck shop menu promotes bad eating habits among students. The majority of participants in this study indicated that students generally purchase lunches and snacks from the tuck shop, and that whatever is available on the menu majorly influences the students’ dietary choices. A New Zealand study [47] indicated that close to two-thirds of students purchased lunches from the tuck shop, which was worrisome because of the high calorie, fat dense and high sugar food options. Another study [48] assessed the compliance of Australian schools by state and territory to set policies guiding the tuck shop menu; the findings indicated that Western Australia was the most compliant with 62% of menu items qualifying for healthy choices, while Queensland was in the bottom three. In this same study, high schools offered more unhealthy food and at lower cost than healthy salads. Even though most tuck shops are run by private conveners and predominantly profit oriented [46], healthy eating policies should be adhered to. The onus therefore lies with the schools to ensure that whatever is sold in the tuck shops is healthy. It is also imperative that the government ensures consistency in the adherence to regulatory guidelines across schools in Australia for healthier tuck shop options.

The proximity of schools to fast food outlets and unhealthy packed lunches from home by students were also identified as barriers in this study. Grier and Davis [49] established that proximity to fast food outlets has a negative impact on the weight of adolescents, particularly those in urban areas and those from low -socio-economic backgrounds. When healthy food items are available at an affordable price, students are more likely to buy them, but their final decision is likely to be influenced by lots of other factors (such as craving, motivations, taste preferences and emotional needs) besides price. Studies have shown that early-life experiences in family systems that reinforce good dietary habits have a role in promoting healthy eating in future life, and this is considered as one of the fundamental ways of addressing adolescent obesity [50]. It is evident in the current study that unhealthy food is competitively cheaper than healthy food encouraging students to fall into the trap of buying unhealthy food due to its affordability. Government funding is needed for sourcing healthy food items and making them available locally, particularly to disadvantaged populations. Constant review of policies governing health promotion in schools is required. This can be made possible through government-regulated food policies as rightly highlighted by participants in this study.

With regards to physical activity, there has been a growing number of students who attend schools very far from their catchment areas [51]. This study has indicated that this makes active transport impossible as parents must drop off their children to school and pick them afterwards. This finding resonates with other studies and indicates that parents could be concerned about the safety of their children, therefore not encouraging active transport to school [44,52]. This implies that schools will need to maximize their physical activity programs to engage students as much as possible to meet the daily requirements for physical activity. The projected [1] increase in obesity in this age group may even be higher looking at the prevailing rate of COVID-19, which has worsened sedentary lifestyle and increased screen time among adolescents recently [6]. This calls for the government to provide more fitness equipment in schools as well as more bikeways and safe footpaths to encourage students to cycle or walk to school. Queensland weather can be hot; hence, the recommendation in this study that the government could provide funding for more facilities like change rooms and showers for freshening up and changing to fresh clothes on such days for students who cycle to school.

Obviously, students’ access to mobile phones has increased over time and this contributes to the worrisome students’ tendency to replace physical activity with games or activities on their electronic devices [6]. It has been established that peers influence each other in being physically active or inactive [53], highlighting the need to consider the impact of peer support in developing future interventions. It was highlighted in this study that strict rules on screen time during school hours and inclusion of more enjoyable non-competitive physical activities could help curb excessive screen time. This result corroborates findings from previous studies and confirm that to get adolescents to be engaged, activities must be enjoyable and pitched at an appropriate skill set level [54].

Interestingly, the participants noted that unhealthy school tuck shop menu, limited funding and lack of trained staff to facilitate physical activities were more prevalent barriers in public schools compared to independent schools. There are significant differences in funding opportunities between these two entities, as public schools are government-funded institutions, while independent schools are privately funded, and this may be a possible reason for the observed differences. The finding also portrays possible differences in the two school environments, likely with different social classes of students and parents. Environment and SES are certainly major contributors to the prevalence of obesity. Higher SES is usually associated with healthy lifestyle behaviour, while low SES is associated with less leisure time, physical activity and the consumption of nutrient-poor, energy-dense diets [11,13,19]. This result indicates that the government needs to do more to better support public schools in combating adolescent obesity. It is important to note that, despite promising initiatives raised by schools, if funding and resources are not available, this can only lead to failure and disappointment because such initiatives cannot be sufficiently implemented or sustained.

The novel addition of this study to existing literature is the development of a reliable model that proposes a multi-pronged stakeholder collaborative approach in developing targeted strategies that foster a supportive ecosystem in combating adolescent obesity and enhancing the achievement of more generalizable health outcomes. This is where the tripartite collaboration between the government, parents and school is needed. The government’s major contribution would be to promote the development of adequate legislation and ensure its enforcement in order to protect adolescents from the marketing and sale of unhealthy foods. School administrators would need to ensure that appropriate school staff are trained to facilitate fun and engaging physical activities, while parents support their children by role modelling healthy lifestyle choices and monitoring screen time at home.

Student mental health and well-being have been reported to be a major priority in schools with persuasive campaigns to normalize asking for help to deal with mental health issues, with promising results in reducing depression among students [55]. As expressed by participants in this study, the same effort can be exerted towards the prevention of adolescent obesity. With its increasing prevalence, adolescent obesity cannot be continuously relegated to the background [5]. The current social climate of ‘fatphobia’ and use of shame-based communications, particularly within high-income countries, emphasizes the need to implement better support strategies for adolescents with obesity [19]. Adolescents affected by this problem should feel comfortable to get help without feeling any prejudice or discrimination.

### 4.1. Strengths and Limitations

The major strength of this study is the utilization of the views of school stakeholders in a mixed-methods study to develop a model for the prevention of adolescent obesity. However, the findings should be interpreted with caution as the study focuses only on a Queensland, Australia context, which may not be applicable to other settings. Additionally, the development of the model for the prevention of adolescent obesity was primarily based on school stakeholders’ perspectives. The perspectives of students, parents and the government were not explored. Furthermore, the collection of data during COVID-19 restrictions limited the response rate and could have caused sampling bias, as participants were only reachable via online platforms.

### 4.2. Implications for Practice and Recommendations for Future Research

Lack of motivation on the part of students and the importance of health education for teachers, students and parents were raised as major areas for consideration in this study. Therefore, the model developed from this study can be used as a guide to support the development of policies and interventions, such as inclusive physical activities for adolescents with obesity and effective strategies for training private tuck shop conveners in the development of a healthy tuck shop menu. Furthermore, school administrators, parents and the government can leverage participants’ suggestions on better ways of incorporating nutritional programs, physical education and overall health strategies that promote the effective prevention of adolescent obesity. Future research from diverse settings and involving the views of students and parents are warranted to substantiate the findings from this study. Further research is also necessary to aid the development of effective educational interventions.

## 5. Conclusions

Despite being a prevailing public health concern that needs to be addressed, adolescent obesity seems to be overlooked as compared to other health problems such as mental health. There are many factors that are at play in dealing with adolescent obesity. The barriers encountered at different stakeholder levels need to be specifically addressed. A tripartite collaboration between all stakeholders is key to effectively addressing adolescent obesity. Practical strategies focusing on nutrition, physical activity and overall health can be employed to improve health outcomes for adolescents. Collaborative stakeholder engagement could include parental education on health, formal health talks in schools by health professionals and better-targeted government funding on advertisements encouraging healthy lifestyle choices. These strategies are instrumental to complementing efforts already made by the school despite its current challenges, which include grappling with a crowded curriculum and limited funding for health promotion interventions against adolescent obesity.

## Figures and Tables

**Table 1:** Demographic profile of participants for the quantitative phase of the study (*n* = 60).

Variable	Category	Frequency	Percent (%)
Gender	Male	13	22
	Female	47	78
School Type	Independent	18	30
	Public	42	70
Role	Head of Department	5	8
	Senior Teacher	3	5
	Teacher	52	87
Education	Bachelor’s degree	38	63
	Master’s degree	15	25
	Doctorate degree	4	7
	Technical college	2	3
	Associate degree	1	2
Employment	Full-time	40	67
	Permanent part-time	14	23
	Contract	4	7
	Daily relief/supply	2	3

**Table 2:** Queensland school stakeholders’ perceptions and beliefs of the causes, enabling and hindering factors of adolescent obesity (*n* = 60).

Construct	Top 3 Agreement Responses and the Least Agreed Response	Frequency	Percent (%)
Causes of adolescent obesity	Poor eating behaviours	59	98
	Sedentary lifestyle	56	93
	Excessive calorie consumption	55	93
	Peer pressure	23	39
Stakeholder beliefs of adolescent obesity	Youth obesity is becoming more prevalent	52	87
	Healthy weight is very important to health of youth	52	87
	Adolescent obesity is a significant cause of peer rejection	41	68
	Only youth who are likely to succeed in a weight loss program should be part of a treatment	2	3
Enabling factors for preventing adolescent obesity in schools	Parental support	51	86
	Regular evaluation of interventions in place	51	86
	Elimination of ‘junk’ food machines	51	86
	Low-calorie healthy lunches should be available during lunch hour	50	86
	Easily accessible fitness equipment during recess	47	78
	Supportive government	47	78
	Community involvement	39	65
Barriers to preventing adolescent obesity in schools	Busy timetable	49	82
	Shortage of trained staff	47	78
	Insufficient program funding	47	78
	The intervention programs are not thoroughly implemented	45	75
	Insufficient motivation of learners to participate in obesity prevention programs	42	70
	Short school day	19	32
Health priorities in schools	Emotional and mental health	54	90
	Relational and social skills	29	48
	Physical fitness	24	40
	Tobacco use	1	0.02

**Table 3:** Interventions and strategies currently used in Queensland schools for physical health and wellness as reported by school stakeholders.

Currently Offered School-Based Interventions	Number of Responses	Percentage (%)
Health and physical education (HPE)	47	78
Other physical activity approaches	27	45
Nutrition education and promotion	21	35
School garden	15	25
Nutrition standards for school meals	7	12
BMI tracking and reporting	1	2
Others	2	3

**Table 4:** Demographic profile of participants for the qualitative phase of the study (*n* = 14).

Participant Name *	Gender	Age Range	Qualification	Role	Years of Experience	Type of High School
1 Angelica	Female	40–45	MS, First Degree	Subject Coordinator, Teacher	10	Public
2 Carol	Female	40–45	First Degree	Teacher	19	Public
3 Chantelle	Female	35–40	First Degree	Teacher	3	Independent
4 Hebron	Male	45–50	First Degree	Teacher	25	Independent
5 Janelle	Female	25–35	MS, First Degree, Grad Cert	Teacher	3	Independent
6 Jesse	Male	35–40	PhD, First Degree, Post Grad Dip	Teacher	3	Independent
7 Jessica	Female	60–65	First Degree	Teacher	50	Public
8 Raphael	Male	55–60	First Degree	Teacher	12	Public
9 Rebecca	Female	35–40	First Degree	Teacher	3	Public
10 Ruth	Female	55–60	MS, First Degree	Teacher	19	Independent
11 Sage	Female	45–50	MS, First Degree	Teacher	13	Public
12 Samantha	Female	45–50	First Degree	Teacher	33	Public
13 Sonia	Female	35–40	MS, First Degree	Teacher	14	Independent
14 Vanya	Female	45–50	First Degree	Teacher	15	Public

* Pseudonyms.

**Table 5:** Triangulation of findings from both the quantitative and qualitative phases of the study.

Stakeholder	Main Themes	Quantitative Findings	Qualitative Findings *	Synthesis of Findings
Parents	Barriers	Poor eating behaviour was indicated by 98% of participants as a hindrance to preventing adolescent obesity.	*‘I know with adolescents comes decreased supervision on what they’re doing and that could potentially impact on the choices they make and what they eat.’ Janelle, PS* ‘*Parental support is important because if parents habitually indulge in junk food, children are going to mimic that, and they are going to think it’s fine and the pattern will be hard to remedy in later life.’, Ruth, IS*	Laxity on the part of parents in supervising and modelling healthy dietary lifestyles can encourage poor eating habits among adolescents.
		Sedentary lifestyle was indicated as a major barrier by 93% of the participants.	*‘I think that unmonitored screen time mostly is even more rampant at home than at school because kids sit down and play video games or go on social media and whatever they like if parents do not keep an eye on them’ Jesse, IS*	Parents’ failure to monitor screen time at home encourages sedentary lifestyle.
	Stakeholder collaboration	Parental support was viewed as a major enabling factor by 86% of the participants.	*‘I think parents who eat healthy and are more active can model the behaviour for their children.’ Rebecca, PS*	Parents have a huge role to play in setting good examples for their children by role-modelling healthy lifestyle, which includes limiting sedentary activities encouraging healthy eating and physical activity.
	Enabling strategies to improve outcome	Participants (86%) indicated that healthy lunches from home would help improve health of adolescents.	‘*I think educating parents would help because you find students coming to school in the morning, they’re drinking soda or coke, as part of breakfast, and that’s not healthy.’ Raphael, PS*	This emphasizes the importance of educating parents about healthy lifestyles and how to be better role models to their adolescent children.
Students	Barriers	Sedentary lifestyle was indicated as a barrier by 93% of the Participants.	‘*During lunch breaks students just sit lazily and go on their phones’, Carol, PS* *‘When students sit around, and they are watching things or playing games on the screen, they are not actively involved in anything, they are not burning off the calories, it does contribute to obesity, and we see many students at lunchtime not playing enough, not running around enough not going to the oval for example and kicking a ball’, Sonia, IS* *‘Students prefer to play games on computers during breaks than get up to play [physical activities]’ Chantelle, IS*	There is a high tendency for students to spend long hours on electronic devices which prevents them from being active during break times.
		Insufficient motivation of learners to participate in obesity prevention programs was indicated as a barrier by 70% of the participants.	*‘The kids are not too bothered to participate, or a bit lazy maybe’, Rebecca, PS*	Students are generally unwilling to participate in programs due to lack motivation. To enhance student motivation, obesity intervention programs should include captivating, engaging and fun physical activities.
		Poor eating behaviour was indicated by 98% of participants as a hindrance to preventing adolescent obesity.	*‘Certainly, you see students in the playground with these big bottles of coke and big family sized packets of potato chips like 200-g packs, instead of the little 20-g, individual serve packs. I even see many with whole packet of biscuits that’s meant to serve eight or 10 people, and they’re eating that. Quite often the kids have part time jobs, or they’ve got pocket money, and they can buy whatever they choose to.’ Jessica, PS*	The tendency for students to indulge in the wrong choice of food is a challenge in dealing with adolescent obesity.
	Enabling strategies to improve outcome	Physical activity was reported as a priority by 40% of the participants.	‘*I think one of the things that will help promote physical activity is not to make all sports competitive; to let students play just for the fun of it.’ Raphael, PS*	There is room for increased student engagement in physical activities. Teachers need to minimize focus on only excelling students and be more inclusive by involving all students in a more enjoyable manner.
School	Barriers	A busy timetable was cited as a major barrier by 82% of the participants	*‘Ideally physical activity should be several times a week, but the crowded curriculum makes it impossible.’ Rebecca, PS* *‘Because it’s a voluntary activity, it depends on whether teachers are free and willing to do it.’ Carol, PS*	The school timetable seems to be too busy to accommodate more physical activity unless there are volunteers who could facilitate such activities outside working hours.
		Shortage of trained staff was reported as a barrier by 78% of the participants	*‘It depends on if teachers are free and willing to do it. I mean, perhaps the school can give more guidance about it’, Carol, PS* *‘If there could be enough trained HPE teachers, trained nutritionists in the schools, that contributes in a positive way to prevent adolescent obesity’, Jesse, IS*	This finding implies that though some teachers may want to encourage physical activity, they find it challenging when they do not have the skills for such activities. An additional challenge is that trained teachers are not always available.
		Excessive calorie consumption was indicated by 93% of the participants.	*‘Students are given whatever they want to purchase so they could choose six pieces of pizza, if they wanted to then get handed that.’ Angelic, PS*	The school tuck shop seems to be very liberal with portion sizes or number of servings per student, thereby encouraging excessive calorie consumption.
		Participants (75%) indicated that lack of thorough implementation of intervention programs is a barrier.	*‘I wouldn’t say that if we’re following clear intervention policies that I have been made aware of them’, Janelle, IS*	Lack of thorough implementation of intervention programs reduces their efficacy.
		Participants (86%) indicated the importance of eliminating vending machines in school environments.	*‘I think there’s also a drink machine, where students can get soft drinks and I think that’s run to raise money for a program of some kind, but it doesn’t seem necessary to have the machine.’ Angelica, PS*	If schools get rid of vending machines, it can facilitate control over what students can eat/drink in school.
	Stakeholder collaboration	Regular evaluation of interventions was a major prevention strategy reported by 86% of the participants.	*‘I don’t remember any staff meeting I’ve ever been to, that’s focused on how to get messages to the students on healthy living or how to model the lifestyle’ Jessica, PS*	Regular evaluation of the efficacy of school-based interventions will facilitate the improvement of functional ones and the disestablishment of ineffective ones.
		Physical fitness was indicated as a school priority by 40% of the participants as compared to emotional and mental health, which was reported as a priority by 90% of the participants.	*‘I think there’s been a lot done within Australia to create awareness of where students can go to get help for depression and if they’re struggling, stigmatized or being bullied, I think, more than ever, over the last 10 years. But I don’t know if we are doing enough in the same respect for adolescent obesity and physical exercise’, Samantha, PS* *‘Zoom into adolescent obesity as a problem. Get it flagged as a problem’ Jesse, IS*	Adolescent obesity needs to be prioritized just like other health concerns that adolescents grapple with.
	Enabling strategies to improve outcome	86% of the participants felt that low-calorie healthy lunches should be available during lunch hour.	*‘Some schools have banned certain items like chips, candies or soft drinks. The number of healthy items they sell at the school tuck shop is quite low in comparison to the number of unhealthy items.’, Angelica, PS* *‘Another way is to make deliberate effort to provide healthy options at the canteen’, Jesse, IS*	Good nutrition strategy for improvement includes targeted efforts to improve the tuck shop menu. Boundaries need to be set on what can or cannot be sold.
Government	Barriers	Insufficient funding for intervention programs was thought to be a barrier by 78% of the participants.	‘*I think schools are trying to do the best we can with very limited funding’ Janelle, IS.* ‘*Lack of funding for those activities that could prevent adolescent obesity is a challenge’, Jesse, IS*	This implies that though schools could develop health promotion activities to tackle adolescent obesity; effective implementation of such activities is mostly hindered by lack of funding.
		Excessive calorie consumption indicated as a barrier by 93% of the participants.	*‘Students habitually walk across the road to purchase big serves of junk food like fries and soft drinks at a fast-food joint near the school’, Sonia, IS*	This shows that some schools are located near fast food outlets, which increases the amount of ‘junk’ food students would normally consume.
		Presence of ‘junk’ food machines in schools was indicated by 86% participants as a major inhibiting factor.	*‘I think there’s also a drink machine, where students can get soft drinks and I think that’s run to raise money for a program of some kind, but it doesn’t seem necessary to have the machine.’ Angelica, PS*	Fund raising using junk food and unlimited purchases from vending machines inhibits progress in preventing adolescent obesity.
		Lack of thorough implementation of intervention programs as reported by 75% of participants reduces the amount of support available to enhance efficacy of the programs.	‘*It is not clearly stated in their roles as teachers, they do not necessarily feel the burden to deal with adolescent obesity’, Jesse, IS* *‘I think there has been a lot done within Australia to create awareness of where students can go to get help for depression and if they’re struggling, stigmatized or being bullied, I think, more than ever, over the last 10 years. But I don’t know if we’re doing enough in the same respect for adolescent obesity and physical exercise’, Samantha, PS*	From the participants’ perspective, mental health has thrived because government-supported programs are put in place while adolescent obesity is not seen as a burden; hence, the lack of unified efforts in dealing with it as a national health concern.
	Stakeholder collaboration	Supportive government was indicated by 78% of the participants.	*‘The government can pay the media to advertise healthy food instead of junk food.’ Ruth, IS* ‘*I think a lot of that is dictated by government regulations. I remember a few years ago they categorized food into red and yellow and green I’m not sure if it’s followed in this school but I’m pretty sure that there has to be regulations they follow as to what they sell at the school, as well as for prevention of obesity.’ Samantha, PS*	This implies that the government can positively influence/regulate school food policies and mandate advertisement of healthy foods.
	Enabling strategies to improve outcome	Easy access to fitness equipment during recess was indicated by 78% of participants as an enabler.	*‘The government can provide funding and fitness equipment, showers and changing rooms for those who cycle to freshen up.’ Jessica, PS*	The implication is that if the government invests in the provision of easily accessible fitness equipment in schools, this could increase students’ interest and involvement in physical activity.

* Illustrative quotes from participants are presented in italics.

## Data Availability

The dataset supporting the findings in this study are included within the article.

## Appendix A. Interview Questions

1. Can you start by briefly elaborating on the role your school plays in the prevention of adolescent obesity?
2. What are some of the general barriers experienced by your school in preventing adolescent obesity?
3. Explain briefly your perception of factors that enable schools in promoting the prevention of adolescent obesity.
4. How can you as a school stakeholder be involved in preventing adolescent obesity?
5. Do you think schools are doing enough to prevent adolescent obesity? Explain further.
6. What are the adolescent obesity prevention interventions and policies developed by your school?
7. How did your school develop these policies and interventions?
8. How effective are the interventions and policies in your school? Elaborate further.
9. Are all student year levels taking part in interventions or is participation optional? Can you discuss ways you think participation can be maximized?
10. On a scale of 1–10 (1 = not implemented and 10 = fully implemented), what is the implementation rate of these policies/interventions at your school? Please justify your rating.
11. Whose responsibility is it to ensure that the interventions and policies in place are implemented and why?
12. Are there policies governing the school tuck shop menu? Please elaborate.
13. What are your recommendations for preventing adolescent obesity?
